# Construction and validation of neonatal sepsis diagnostic nomograms: a single-site retrospective study

**DOI:** 10.3389/fped.2026.1841983

**Published:** 2026-07-28

**Authors:** Yun Yu, Huawei Wang, Fengxia Huang, Xin Ding

**Affiliations:** Neonatology Department, Children’s Hospital of Soochow University, Suzhou, China

**Keywords:** diagnostic prediction model, internal validation, LASSO, logistic regression, neonatal sepsis, neonatal septicemia, nomogram, ROC curve

## Abstract

**Background:**

Neonatal sepsis remains a major cause of morbidity and mortality, while blood culture, although considered the diagnostic reference standard, is limited by delayed reporting and reduced sensitivity in routine practice. This study aimed to develop and internally validate practical diagnostic nomograms for neonatal sepsis using routinely available clinical and laboratory data.

**Methods:**

We conducted a single-center retrospective study using medical record and laboratory data from the Children's Hospital of Soochow University between October 13, 2019 and October 13, 2025. After screening 523 records, 483 neonates with sepsis were included in the case group, and controls were sampled from hospitalized neonates with non-septic infection or no infection during the same period. Candidate predictors were screened using univariable logistic regression and further reduced using least absolute shrinkage and selection operator regression, with final variable selection informed by clinical relevance and published evidence. Two logistic regression models were constructed and presented as nomograms: model A included maternal and infant clinical variables, and model B additionally incorporated routine laboratory indicators. Internal validation was performed using a 7:3 training-test split. Model performance was assessed by discrimination and calibration.

**Results:**

The training and test sets included 338 and 145 sepsis cases and 140 and 60 controls, respectively. Model A incorporated maternal residence, threatened abortion history, breastfeeding status, prenatal antibiotic exposure, infant temperature, weak mental response, primitive reflex status, antibiotics before visit, and weight difference between visit and birth. Model B added abnormal white blood cell count and elevated C-reactive protein. In the training and test sets, the area under the receiver operating characteristic curve was 0.839 and 0.852 for model A, and 0.905 and 0.923 for model B, respectively. Sensitivity and specificity were 0.654 and 0.907 for model A in the training set and 0.710 and 0.867 in the test set; corresponding values for model B were 0.864 and 0.800 in the training set and 0.910 and 0.750 in the test set. Calibration was acceptable overall, although model B showed weaker calibration in the training set.

**Conclusion:**

Two nomogram-based diagnostic models for neonatal sepsis were developed and internally validated in a single-center retrospective case-control sample. Both models showed useful discriminatory ability, and the model combining clinical and laboratory variables performed better. Because the nomogram probabilities are sample-based estimates rather than absolute population risks, external validation and prevalence-based recalibration are needed before broader clinical application.

## 1 Introduction

Neonatal sepsis remains a paramount global health challenge, constituting a leading cause of morbidity and mortality in the first month of life. Worldwide, it is estimated to affect approximately 2,824–3,930 cases per 100,000 live births, contributing significantly to the nearly 50% of under-five deaths attributed to the combination of sepsis and preterm birth (1, 2). The burden is disproportionately shouldered by low- and middle-income countries (LMICs), where the majority of neonatal deaths occur (2). In China, although age-standardized mortality rates for neonatal disorders have shown a commendable decreasing trend since 1990, the absolute number of incident cases remains substantial, and neonatal sepsis continues to be a major specific cause within this category (3, 4). The clinical imperative for prompt recognition and treatment is unequivocal, as delays can rapidly precipitate multisystem organ failure and death (5, 6). This urgency is compounded by the disease's frequently non-specific and subtle initial presentation, which poses a fundamental diagnostic dilemma for clinicians (5, 7).

The clinical manifestation of neonatal sepsis is notoriously insidious, often overlapping with non-infectious transitional states common in the newborn period. Signs such as temperature instability, respiratory distress, feeding intolerance, and altered activity level are neither sensitive nor specific, making the distinction between infected and uninfected infants exceptionally difficult based on clinical grounds alone (5, 8). This diagnostic uncertainty, coupled with the high stakes of missing a true infection, creates a clinical environment prone to the over-investigation and empiric treatment of a large number of well-appearing or mildly symptomatic infants (9, 10). Consequently, a significant proportion of neonates worldwide are exposed to antibiotics for suspected sepsis, a practice that carries its own risks, including the promotion of antimicrobial resistance, alteration of the developing microbiome, and increased healthcare costs (5, 11).

For decades, the microbiological confirmation of neonatal sepsis has relied on blood culture, which is conventionally regarded as the diagnostic reference standard (12). This method, however, is fraught with significant limitations that undermine its practical utility in guiding urgent clinical decisions. The turnaround time for definitive culture results typically exceeds 24–48 h, a critical delay when therapeutic decisions must be made within hours of symptom onset (13, 14). Furthermore, its sensitivity is suboptimal, often reported below 50%, due to several intrinsic factors: the low volume of blood that can be safely drawn from a neonate, particularly from extremely low birth weight infants; prior administration of intrapartum or postnatal antibiotics which can sterilize the bloodstream; and the fastidious nature of some pathogens (12, 15, 16). The phenomenon of “culture-negative” sepsis is thus widespread, leading to diagnostic ambiguity and heterogeneous case definitions that complicate both clinical management and epidemiological surveillance (17, 18).

In light of these limitations, clinicians have long sought rapid adjunctive tools to aid in the early diagnostic triage of suspected sepsis. Routine hematological indices and inflammatory biomarkers, such as the white blood cell count (WBC), C-reactive protein (CRP), procalcitonin (PCT), and interleukin-6 (IL-6), are widely employed (19, 20). Systematic assessments reveal that PCT may offer better early correlation with proven sepsis than CRP, yet no single biomarker achieves sufficient diagnostic accuracy to rule in or rule out infection independently (11, 16, 21). CRP is particularly valued for its high negative predictive value in serial measurements, helping to confidently discontinue antibiotics in clinically stable infants (22, 23). Combinations of biomarkers, such as PCT with CRP or IL-6, have demonstrated improved diagnostic performance over single markers, but their utility is still constrained by variable cut-off values influenced by gestational age, time of sampling, and non-infectious inflammatory conditions (19, 24, 25). Therefore, while helpful in conjunction with clinical assessment, biomarkers alone cannot resolve the core diagnostic uncertainty (19, 26).

This landscape of diagnostic imperfection has catalyzed a paradigm shift towards multivariable clinical prediction models. The integration of multiple predictor variables, encompassing maternal risk factors, perinatal history, clinical signs, and laboratory parameters, into a single, quantifiable risk score offers a more nuanced and potentially more accurate assessment than any isolated finding (27, 28). In pediatrics and neonatology, the development and application of such models, often visualized as user-friendly nomograms, represent a move towards more personalized and evidence-based decision-making (29, 30). Nomograms translate complex statistical models into practical bedside tools that can estimate the probability of a disease outcome for an individual patient, facilitating communication and clinical utility (31).

Several prediction tools for neonatal sepsis have been developed, reflecting different clinical intents and settings. The most prominent example is the early-onset sepsis (EOS) calculator, a risk-stratification tool designed for term and late-preterm infants in the first 72 h of life. It incorporates maternal risk factors and infant clinical examination to guide management, and its implementation in various settings has demonstrated a significant reduction in unnecessary antibiotic use and laboratory testing for EOS (10, 32, 33). However, its scope is intentionally narrow, targeting only early-onset disease. For the broader population of hospitalized neonates, including those with late-onset sepsis (LOS) and those admitted to neonatal intensive care units (NICUs) with evolving symptoms, a universally applicable diagnostic tool is lacking. Existing models for hospital-based neonatal sepsis diagnosis show considerable heterogeneity in their predictor variables, often rely on laboratory parameters that may not be immediately available in all settings, and have frequently been validated only in specific, often high-risk, cohorts within single healthcare systems (27, 28, 34, 35). Many models incorporate advanced or specialized biomarkers not routinely available at the point of care, limiting their generalizability, especially in resource-constrained environments where the sepsis burden is highest (6, 27).

There exists, therefore, a clear unmet need for a pragmatic, broadly applicable diagnostic model that leverages data routinely and rapidly available at the bedside of any sick neonate. A model predicated on easily obtainable maternal and perinatal history, fundamental clinical manifestations noted during physical examination, and basic laboratory tests like complete blood count and CRP could bridge a critical gap. Such a tool would not aim to replace clinical judgment or blood culture but to augment the clinician's ability to stratify risk more accurately at the time of initial evaluation, potentially informing future, more judicious use of empiric antibiotics and diagnostic resources after external validation (28, 36). Furthermore, exploring the incremental value of adding routine laboratory data to a model based solely on clinical features could inform tiered diagnostic approaches suitable for different levels of healthcare infrastructure.

Despite the proliferation of sepsis prediction research, a notable gap persists in the development and rigorous internal validation of practical diagnostic nomograms specifically designed for the undifferentiated neonatal inpatient population, utilizing the comprehensive electronic medical record data available in modern hospital systems. Prior studies have often focused on specific subgroups, relied on limited variable sets, or prioritized prognostic over diagnostic prediction (37, 38). Consequently, this study was designed to address this gap by developing and internally validating two logistic-regression-based diagnostic nomograms for neonatal sepsis. The first model (Clinical Model) utilizes primarily maternal, perinatal, and infant clinical features, while the second (Clinical-Laboratory Model) additionally incorporates readily available routine laboratory indicators. By comparing the performance of these models, this single-center retrospective study aims to provide a practical, evidence-based tool to assist in the timely and accurate diagnosis of neonatal sepsis in a hospital setting.

## 2 Materials and methods

### 2.1 Study design

This study was a retrospective case-control study, aiming to optimize the diagnostic methods of neonatal septicemia, and complete the construction of the diagnostic prediction model. The study was based on real-world data from the medical record system and the laboratory system, adopted a case group and control group design, and completed the development and internal validation of the model. Because the proportion of sepsis cases was determined by sampling rather than by the true prevalence among all neonates evaluated for suspected sepsis, predicted probabilities generated by the nomograms should be interpreted as sample-based risk estimates and should not be considered absolute clinical probabilities unless recalibrated to the target population prevalence.

### 2.2 Study site and study period

The study site was the Children's Hospital of Soochow University. The study time range was from October 13, 2019 to October 13, 2025. The Children's Hospital of Soochow University is a tertiary pediatric referral center in Suzhou, China. Study subjects were neonates admitted to the neonatal intensive care unit (NICU) or the general neonatal ward. Infants were referred from birth hospitals, outpatient clinics, or pediatric emergency departments in the Suzhou metropolitan area and surrounding regions. Neonates evaluated exclusively in well-baby nurseries or postnatal wards were not included in the study population.

### 2.3 Study subjects and sample size

**Figure 1:**
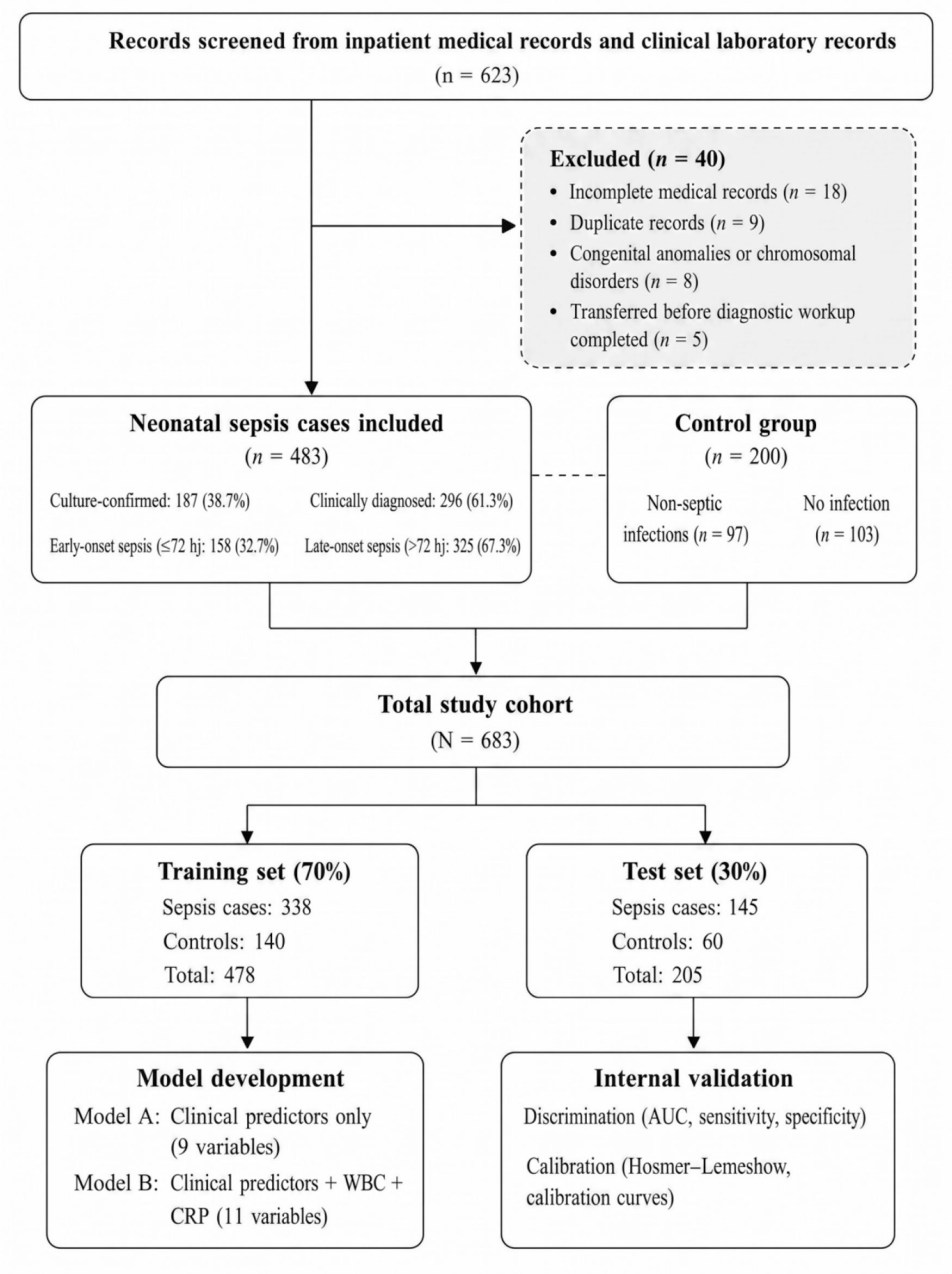
Study flowchart illustrating cohort assembly, case and control selection, and allocation to training and test sets. A total of 523 records were screened from inpatient medical records and clinical laboratory records at the Children's Hospital of Soochow University (October 2019–October 2025). After exclusion of 40 records, 483 neonatal sepsis cases were included, of which 187 (38.7%) were culture-confirmed and 296 (61.3%) were clinically diagnosed. By onset timing, 158 (32.7%) were classified as early-onset sepsis (EOS, ≤72 h of life) and 325 (67.3%) as late-onset sepsis (LOS, >72 h). The control group comprised 97 neonates with non-septic infections and 103 non-infected neonates hospitalized during the same period (*n* = 200). The total cohort (*n* = 683) was divided by stratified random sampling into a training set (70%; 338 cases, 140 controls) and a test set (30%; 145 cases, 60 controls). Model A was constructed using nine clinical predictors without laboratory tests; Model B augmented these with white blood cell count and C-reactive protein (11 predictors). Internal validation assessed discrimination (area under the receiver operating characteristic curve, sensitivity, specificity) and calibration (Hosmer–Lemeshow test, calibration curves). AUC, area under the curve; CRP, C-reactive protein; WBC, white blood cell count.

The study subjects were retrieved through inpatient medical record records and clinical laboratory records. A total of 523 related cases were screened, after data processing 483 valid septicemia cases were obtained and were included in the case group analysis and modeling. The control group comprised 200 neonates sampled from the same institution during the study period: 97 neonates hospitalized with non-septic infections (such as localized skin infections, urinary tract infections, or pneumonia not meeting sepsis criteria) and 103 neonates hospitalized for non-infectious conditions (such as neonatal jaundice, feeding difficulties, or observation following perinatal events) with no clinical or laboratory evidence of infection. This mixed control group was chosen to reflect the spectrum of clinical comparisons encountered in routine neonatal practice. A study flowchart illustrating cohort assembly is provided in Figure 1.

### 2.4 Inclusion criteria and exclusion criteria

The inclusion criteria were implemented with reference to the diagnostic plans and clinical diagnosis and treatment specifications related to neonatal septicemia. Septicemia cases needed to meet the requirements of bacteriological etiological diagnosis or clinical diagnosis for inclusion, that was, positive clinical manifestations and blood culture positive, or there were positive clinical manifestations and 2 or more abnormalities among the non-specific laboratory examination indicators. The exclusion criteria included incomplete case data, admission age greater than 28 days, and those with clearly severe cardiovascular disease, genetic metabolic disease, congenital developmental malformations, familial genetic disease, familial immune disease, recent surgery and other conditions. During data processing, those with severe missing key variables, diagnostic information that could not be verified, or obvious logical conflicts were excluded.

### 2.5 Outcome variable definition

The outcome variable of this study was whether neonatal septicemia occurred (yes/no). The determination of septicemia was determined comprehensively based on etiological evidence and clinical diagnostic standards, and the outcome determination was based on the final diagnosis during hospitalization, and was checked together with blood culture results, clinical manifestations and laboratory examination records.

For subgroup analyses, early-onset sepsis (EOS) was defined as sepsis with onset within the first 72 h of life, and late-onset sepsis (LOS) was defined as sepsis with onset after 72 h of life. These definitions were applied based on the documented postnatal age at the time of initial clinical presentation or blood culture collection, consistent with widely used classification criteria in neonatal infectious disease literature.

### 2.6 Positive clinical manifestations and laboratory abnormality determination

Positive clinical manifestations included unstable body temperature, fever or no rise in body temperature could occur. For model coding, infant temperature was classified as abnormal when axillary temperature was ≥37.5 °C or <36.5 °C at admission or during the initial sepsis evaluation; values within 36.5–37.4 °C were classified as normal. Axillary temperature measurement was used per institutional protocol owing to its established safety, practicality, and widespread adoption in Chinese neonatal units. Although rectal temperature measurement remains the reference standard for core body temperature assessment in neonates, axillary measurement was preferred in this setting to minimize procedural discomfort and infection risk. The selected thresholds (≥37.5 °C and <36.5 °C) are consistent with commonly used axillary reference ranges in Chinese neonatal clinical guidelines. Temperature was assessed at the time of admission or during the initial clinical evaluation for suspected sepsis, using standardized digital thermometers with a minimum measurement duration of 3 min in the axillary position. Other positive clinical manifestations included the respiratory system could have manifestations such as groaning, shortness of breath, cyanosis, weak crying, the circulatory system could manifest as changes in heart rate and heart rhythm and poor perfusion, the digestive system could manifest as refusal to feed, vomiting, abdominal distension, diarrhea, jaundice, etc., the nervous system could manifest as weak mental response, lethargy, poor response, etc., the hematologic system could be due to thrombocytopenia and/or abnormal coagulation function and showed bleeding tendency, the urinary system could have oliguria or anuria and other conditions, in addition the primary infection focus could have manifestations such as skin pustules and ulceration. The determination of non-specific laboratory examination abnormalities was implemented according to commonly used clinical thresholds, mainly including abnormal white blood cell count and classification, abnormal I/T, elevated C-reactive protein, elevated procalcitonin, elevated PCT, decreased platelet count, elevated erythrocyte sedimentation rate, etc. In order to exclude the physiological factors on procalcitonin, for neonates within 72 h after birth procalcitonin was not used as the basis for determination.

### 2.7 Data collection and variable acquisition

According to the diagnostic standards and literature review results, records of various data of the study subjects were completed, and the data sources included electronic medical records, nursing records, laboratory systems, imaging and microbiology system records. The collected contents covered neonatal general information and perinatal information, neonatal mother information, perinatal and medical history information, clinical manifestations, laboratory examinations, etiological and drug susceptibility information, etc. Neonatal general information included gender, admission age, gestational age, admission weight, birth weight, etc., mother information included age, residential area, pregnancy diseases, conception methods, prenatal medication and prenatal antibiotic use, etc., perinatal and medical history information included gestational weeks, pregnancy times and parity, delivery mode, amniotic fluid placenta and umbilical cord conditions, intrauterine distress conditions, postnatal asphyxia and resuscitation conditions, feeding mode, infection contact history, pre-admission treatment conditions, etc. Clinical manifestations included body temperature, respiration, heart rate, symptoms and positive physical signs. Laboratory examinations included whole blood cell count and classification, band neutrophil ratio, C-reactive protein, procalcitonin, blood gas analysis indicators, lactate, electrolytes, liver and kidney function, myocardial enzyme spectrum, prealbumin, etc. Etiological and drug susceptibility information included blood culture pathogens, drug susceptibility results, blood culture positive reporting time, and if necessary included cerebrospinal fluid etiological results.

### 2.8 Specimen collection and testing process

Blood specimen collection of study subjects was completed as early as possible after admission, and was collected before antibiotic use, under the premise of strict aseptic operation a venous or arterial blood collection method was used to complete submission for testing. For those clinically suspected to be complicated with central nervous system infection, routine mobilization was carried out, and lumbar puncture cerebrospinal fluid examination was completed under the informed consent of the family members, and the specimens were sent for testing as soon as possible after collection. Laboratory testing was implemented according to the hospital routine process, routine blood, blood gas, biochemistry, immunity and related inflammation indicator testing were all completed by the laboratory department according to standard operating procedures, microbial culture was completed by the microbiology room according to standard process and the positive reporting time was recorded.

### 2.9 Data processing and quality control

Consistency check and logical verification were carried out on the original data, variable units and value ranges were unified, obvious input errors were corrected, and distribution assessment and extreme value checking were performed on continuous variables. Statistical description was performed for missing key variables. Missingness was low for candidate predictors, and complete-case analysis was used for model development and validation; no multiple imputation was performed. The whole research process adopted key variable sampling rechecking and result backtracking checking methods to control information bias and input bias.

### 2.10 Prediction model development and internal validation

Model construction adopted logistic regression method, established a diagnostic prediction model and presented it in the form of a nomogram. Candidate variable screening first performed univariable logistic regression analysis to preliminarily identify variables related to the outcome, then combined clinical significance and literature evidence to determine the candidate variable set, and if necessary used LASSO regression to reduce dimensionality and select non-zero coefficient variables into multivariable modeling. Multivariable modeling used logistic regression to construct the model, categorical variables set reference levels according to clinical significance and performed dummy variable processing, continuous variables were preferentially included in continuous form, and if necessary standardization or reasonable transformation was carried out. Model presentation gave an individualized prediction tool in the form of a nomogram and formed a scoring system that could be used for clinical application. Internal validation used training set and test set splitting according to data scale and event proportion and ensured that the proportion of positive and negative in the training set and test set was as consistent as possible, and at the same time combined bootstrap method to perform model stability assessment and overfitting correction. A single 7:3 random split was chosen as the primary internal validation strategy because the overall sample size (*n* = 683) and event count (*n* = 483 sepsis cases) were considered sufficient to yield stable estimates in both partitions while maintaining clinical interpretability. We acknowledge that bootstrapping or repeated k-fold cross-validation generally provide less partition-dependent performance estimates; however, the single-split approach was preferred here for its simplicity and direct comparability with commonly reported prediction modeling frameworks in neonatal medicine. The training-set and test-set case proportions were balanced by stratified random sampling to ensure comparable outcome distributions across partitions. Model performance evaluation was carried out from two aspects of discrimination and calibration. Discrimination used the area under the ROC curve and reported sensitivity and specificity. Sensitivity and specificity were calculated using probability cutoffs selected in the training set by maximizing the Youden index (sensitivity + specificity - 1); the same cutoffs were then applied to the test set. The selected probability cutoffs were 0.64 for model A and 0.58 for model B. Calibration used the Hosmer-Lemeshow test and calibration curve to evaluate the consistency of predicted probability and actual occurrence probability, and if necessary further reported calibration slope and calibration intercept.

### 2.11 Statistical analysis

Statistical analysis was completed using SPSS22.0 statistical software. Continuous variables were expressed as mean ± standard deviation or median (interquartile range) according to distribution type, categorical variables were expressed as frequency and percentage. Comparison between two groups selected t test, Wilcoxon rank-sum test, chi-square test or rank-sum test according to data type and distribution, multiple group comparison used analysis of variance or Kruskal–Wallis test. Normality test used Shapiro–Wilk method. All tests were two-sided tests, *P* < 0.05 was considered that the difference had statistical significance.

### 2.12 Ethics statement

This study had been approved by the Ethics Committee of the Children's Hospital of Soochow University, approval number 23CS193. The study used retrospective medical records and laboratory data, strictly implemented data de-identification and privacy protection requirements, and the research data were only used for scientific analysis.

## 3 Results

### 3.1 Univariate logistic regression analysis results of clinical data in control group and case group

The clinical data of 200 children in the control group and 483 children in the case group were analyzed by univariate logistic regression analysis, and the results with statistical significance were summarized as shown in Table 1.

**Table 1:** Univariate logistic regression analysis results of clinical data in control group and case group.

Variable	Category	Control group (*N* = 200), *n* (%)	Case group (*N* = 483), *n* (%)	*P*	OR (95% CI)
Maternal residence area	City	122 (61.00)	205 (42.44)		1.000 (reference)
	Rural	78 (39.00)	278 (57.56)	<.0001	2.121 (1.515, 2.970)
Delivery place	Tertiary hospital	100 (50.00)	167 (34.58)		1.000 (reference)
	Secondary hospital	93 (46.50)	299 (61.90)	0.0002	1.925 (1.370, 2.705)
	Primary hospital	6 (3.00)	4 (0.83)	0.1891	0.399 (0.110, 1.449)
	Home or others	1 (0.50)	13 (2.69)	0.0216	7.784 (1.003, 60.410)
Threatened abortion	No	199 (99.50)	455 (94.20)		1.000 (reference)
	Yes	1 (0.50)	28 (5.80)	0.0142	12.246 (1.655, 90.636)
Maternal anemia	No	190 (95.00)	436 (90.27)		1.000 (reference)
	Yes	10 (5.00)	47 (9.73)	0.0458	2.048 (1.014, 4.139)
Prenatal antibiotics	No	195 (97.50)	444 (91.93)		1.000 (reference)
	Yes	5 (2.50)	39 (8.07)	0.009	3.521 (1.369, 9.059)
Breastfeeding	Yes	140 (70.00)	265 (54.86)		1.000 (reference)
	No	60 (30.00)	218 (45.14)	0.0003	1.915 (1.344, 2.730)
Infant temperature	Normal	160 (80.00)	310 (64.18)		1.000 (reference)
	Abnormal	40 (20.00)	173 (35.82)	<.0001	3.191 (2.075, 4.907)
Weak mental response	No	190 (95.00)	348 (72.05)		1.000 (reference)
	Yes	10 (5.00)	135 (27.95)	<.0001	6.129 (3.229, 11.634)
Umbilical stump exudation	No	179 (89.50)	377 (78.05)		1.000 (reference)
	Yes	21 (10.50)	106 (21.95)	0.0713	1.524 (0.964, 2.408)
Primitive reflex	Sound	196 (98.00)	405 (83.85)		1.000 (reference)
	Not sound	4 (2.00)	78 (16.15)	<.0001	13.626 (4.253, 43.659)
Umbilical infection focus	No	184 (92.00)	377 (78.05)		1.000 (reference)
	Yes	16 (8.00)	106 (21.95)	<.0001	5.024 (2.564, 9.843)
Antibiotics before visit	No	184 (92.00)	338 (69.98)		1.000 (reference)
	Yes	16 (8.00)	145 (30.02)	<.0001	6.492 (3.328, 12.661)
WBC abnormal	No	190 (95.00)	338 (69.98)		1.000 (reference)
	Yes	10 (5.00)	145 (30.02)	<.0001	21.021 (7.665, 57.650)
Neutrophils >60%	No	170 (85.00)	328 (67.91)		1.000 (reference)
	Yes	30 (15.00)	155 (32.09)	<.0001	2.576 (1.688, 3.933)
CRP elevated	No	180 (90.00)	251 (51.97)		1.000 (reference)
	Yes	20 (10.00)	232 (48.03)	<.0001	6.744 (4.277, 10.634)
PCT elevated	No	60 (30.00)	106 (21.95)		1.000 (reference)
	Yes	140 (70.00)	377 (78.05)	0.0095	1.612 (1.124, 2.312)

OR referred to 1 vs. 0 or category vs. reference category. Percentages were calculated within group.

Across the candidate predictors evaluated for model development, the proportion of missing values ranged from 0.0% to 4.7%. Missingness for variables retained in the final models ranged from 0.0% to 3.9%, and complete-case records were used for model fitting and validation.

Supplementary analyses stratified by diagnostic confirmation and onset timing demonstrated relatively consistent model performance across subgroups (Supplementary Table S2). When the analysis was restricted to culture-confirmed sepsis cases only, test-set AUC values were 0.831 (95% CI, 0.761–0.901) for model A and 0.876 (95% CI, 0.812–0.940) for model B. In the EOS subgroup, corresponding values were 0.861 and 0.918; in the LOS subgroup, 0.841 and 0.901. Notably, when controls were restricted to the 97 non-septic infection cases, discrimination decreased for both models (model A AUC 0.816; model B AUC 0.889), consistent with the expectation that distinguishing sepsis from other infectious conditions is clinically more challenging than distinguishing sepsis from non-infected neonates.

### 3.2 Construction of neonatal septicemia diagnostic prediction model

The study subjects were divided into training set and test set according to the ratio of 7:3, the samples of case group and control group in the training set were 338 and 140 cases respectively, and the samples of case group and control group in the test set were 145 and 60 cases respectively.

Of the 483 sepsis cases, 187 (38.7%) were culture-confirmed and 296 (61.3%) were clinically diagnosed on the basis of positive clinical manifestations combined with two or more non-specific laboratory abnormalities. Regarding onset timing, 158 cases (32.7%) met the definition for early-onset sepsis (EOS, onset ≤72 h of life) and 325 cases (67.3%) were classified as late-onset sepsis (LOS, onset >72 h of life). The median postnatal age at admission was 5 days (interquartile range [IQR] 2–12 days) in the sepsis group and 4 days (IQR 1–10 days) in the control group (*P* = 0.08). Among sepsis cases, 212 (43.9%) were admitted within the first 72 h of life, during which period procalcitonin was not used as a diagnostic criterion owing to physiological postnatal elevation. The control group comprised 97 (48.5%) neonates with non-septic infections and 103 (51.5%) neonates without infection.

**Figure 2:**
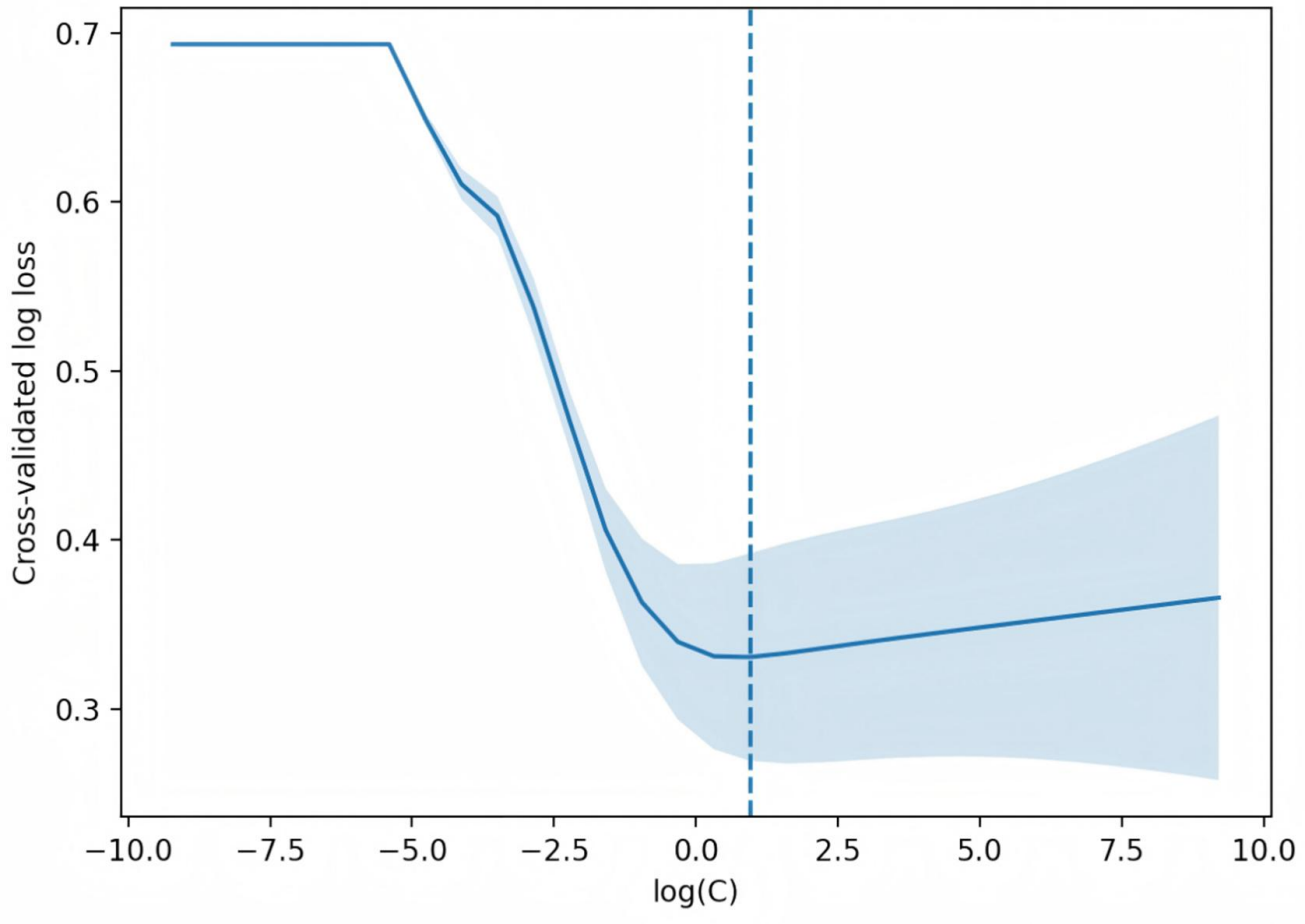
LASSO logistic regression 10-fold cross-validation. LASSO regression was used to reduce the dimensionality of the data. Under different values of the inverse regularization parameter C, the cross-validated log loss and its fluctuation range were evaluated; C denotes the inverse regularization strength used by the software implementation, with larger C indicating weaker penalization. The cross-validation results showed that log(C) = 0.95 corresponded to the smallest log loss, and the coefficients of some variables were compressed to near 0.

**Figure 3:**
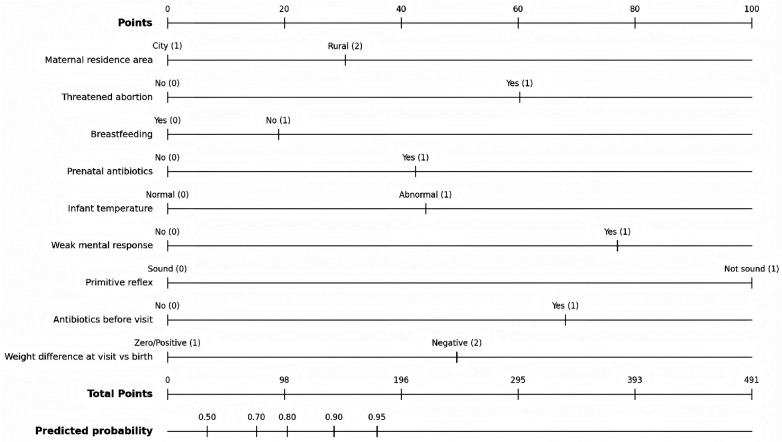
Neonatal septicemia prediction model A. In the figure, the meaning of different items and values was, maternal residence area: 1 was city, 2 was rural; threatened abortion history: 0 was no, 1 was yes; breastfeeding: 0 was yes, 1 was no; maternal prenatal antibiotic treatment: 0 was no, 1 was yes; infant temperature: 0 was normal, 1 was abnormal; weak mental response: 0 was no, 1 was yes; primitive reflex: 0 was sound, 1 was not sound; antibiotics before visit: 0 was no, 1 was yes; weight difference at visit vs. birth: 1 was zero or positive value, 2 was negative value. Because this study used case-control sampling, the predicted probabilities should be interpreted as sample-based risk estimates rather than absolute clinical probabilities unless recalibrated to the target population prevalence.

Based on the variables and combined with literature review and clinical work characteristics, model A and model B were finally established, among them the prediction variables of model A included maternal residence area, maternal threatened abortion history, breastfeeding, maternal prenatal antibiotic treatment, infant temperature, weak mental response, primitive reflex, antibiotics treatment before visit, and weight difference at visit and birth weight a total of 9 variables, mainly involving maternal history and infant clinical manifestations; model B prediction variables on the basis of model A, added laboratory examination indicators that could be completed by medical institutions, that was, peripheral blood white blood cell count abnormal and C-reactive protein elevated. Figures 2, 3 were the nomogram-like plots of model A and model B respectively.

### 3.3 Performance evaluation of neonatal septicemia diagnostic prediction model

Discrimination of the model: the results of Table 2 showed the sensitivity and specificity of model A and model B on the training set and test set. Among them the sensitivity and specificity of model B were higher than model A.

**Table 2:** Sensitivity and specificity of model A and model B at training-set youden-index probability cutoffs.

Evaluation index	Model A training set	Model A test set	Model B training set	Model B test set
Sensitivity	0.654 (95% CI, 0.602–0.703)	0.710 (95% CI, 0.632–0.778)	0.864 (95% CI, 0.823–0.896)	0.910 (95% CI, 0.853–0.947)
Specificity	0.907 (95% CI, 0.848–0.945)	0.867 (95% CI, 0.758–0.931)	0.800 (95% CI, 0.726–0.858)	0.750 (95% CI, 0.628–0.842)

The probability cutoffs were selected in the training set by maximizing the Youden index and were then applied unchanged to the test set: 0.64 for model A and 0.58 for model B. Confidence intervals were calculated using the Wilson method.

**Figure 4:**
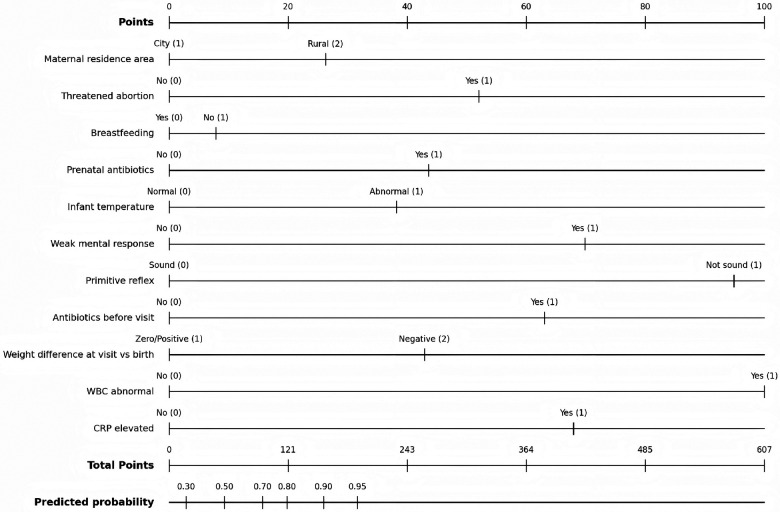
Neonatal septicemia prediction model B. In the figure, the meaning of different items and values was, on the basis of model A, peripheral blood white blood cell count abnormal: 0 was no, 1 was yes; C-reactive protein elevated: 0 was no, 1 was yes. Because this study used case-control sampling, the predicted probabilities should be interpreted as sample-based risk estimates rather than absolute clinical probabilities unless recalibrated to the target population prevalence.

**Figure 5:**
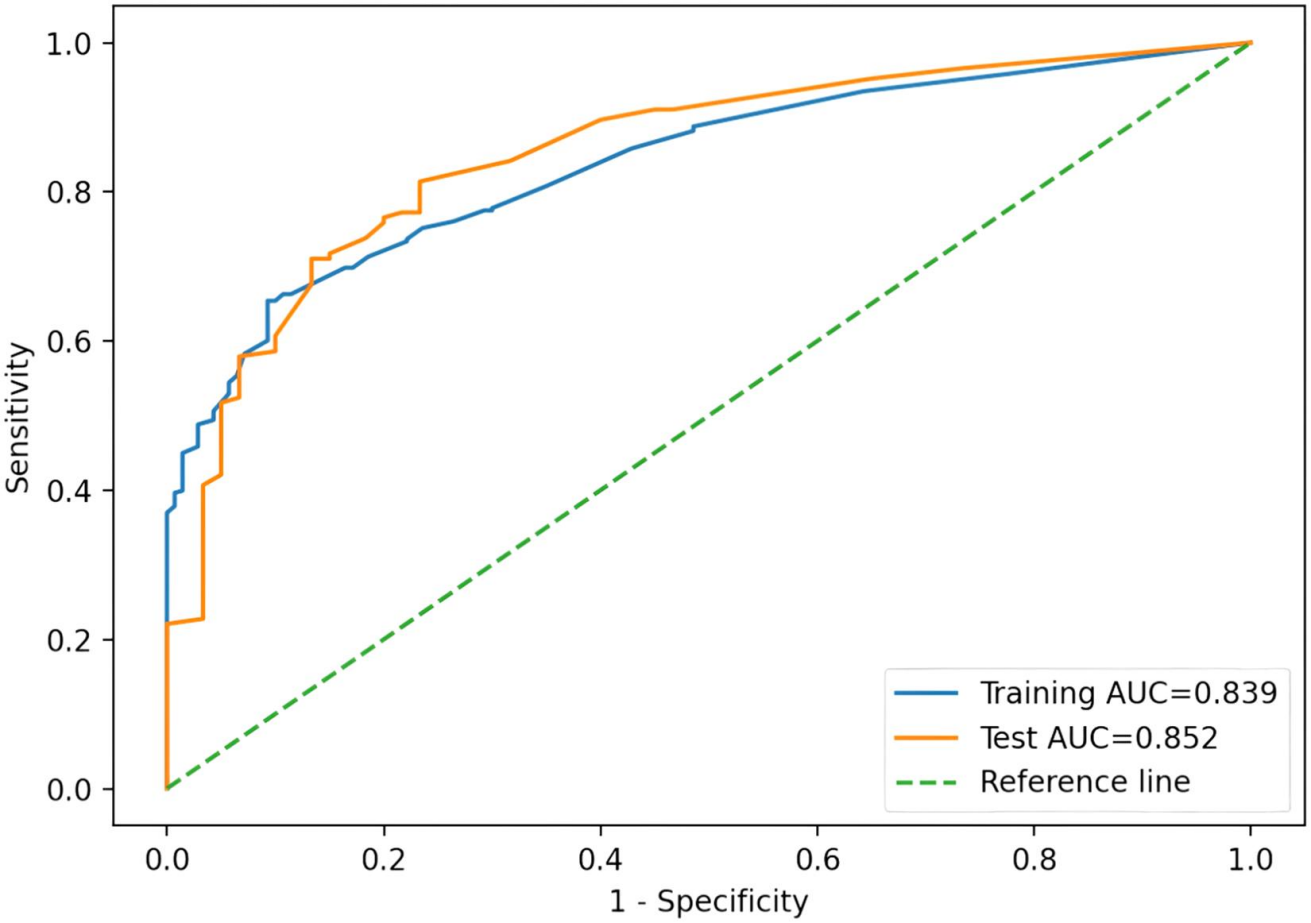
ROC curves of neonatal septicemia prediction model A in training set and test set. The area under ROC curve of the training set was 0.839, and the area under ROC curve of the test set was 0.852.

The results of Figure 4 showed the area under the ROC curve of model A on the training set and test set, which were 0.839 and 0.852 respectively. The results of Figure 5 showed the area under the ROC curve of model B on the training set and test set, which were 0.905 and 0.923 respectively. The area under curve of model B was higher than model A.

### 3.4 Calibration of the model

**Figure 6:**
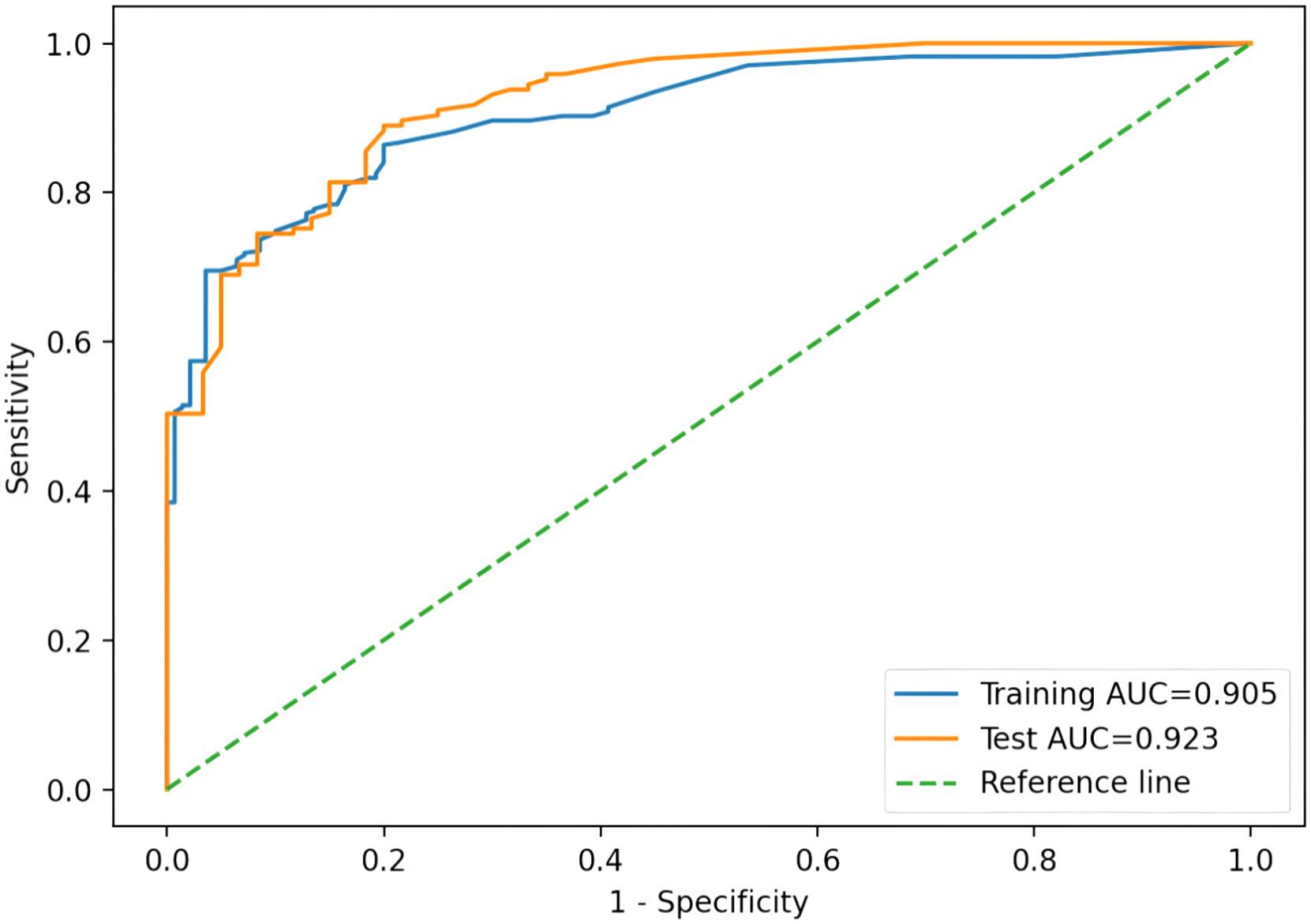
ROC curves of neonatal septicemia prediction model B in training set and test set. The area under ROC curve of the training set was 0.905, and the area under ROC curve of the test set was 0.923.

**Figure 7:**
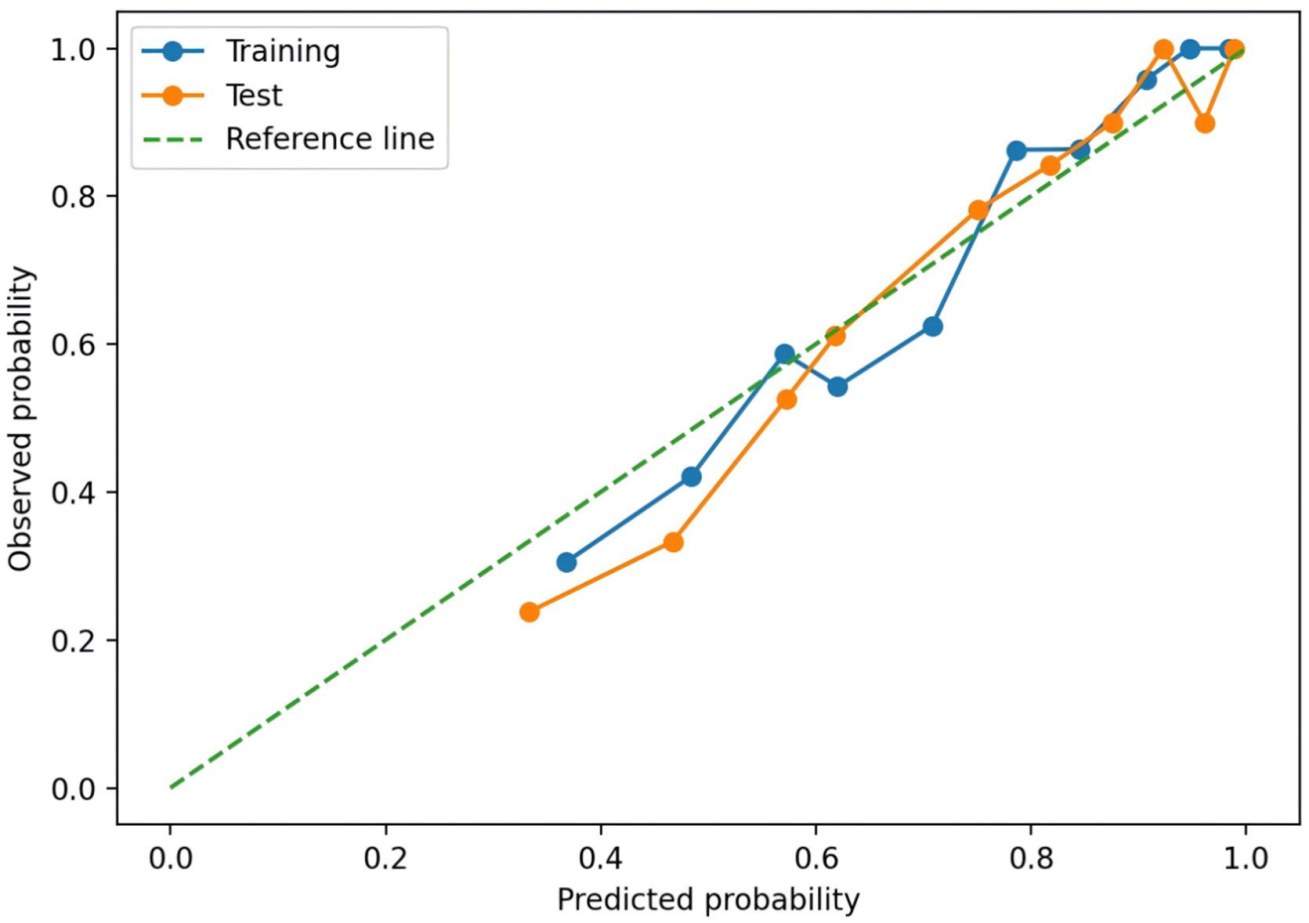
Calibration curves of neonatal septicemia prediction model A in training set and test set. The calibration curves of model A in the training set and test set were close to the reference line, suggesting acceptable consistency between sample-based predicted probability and observed outcome proportion within this case-control sample.

**Figure 8:**
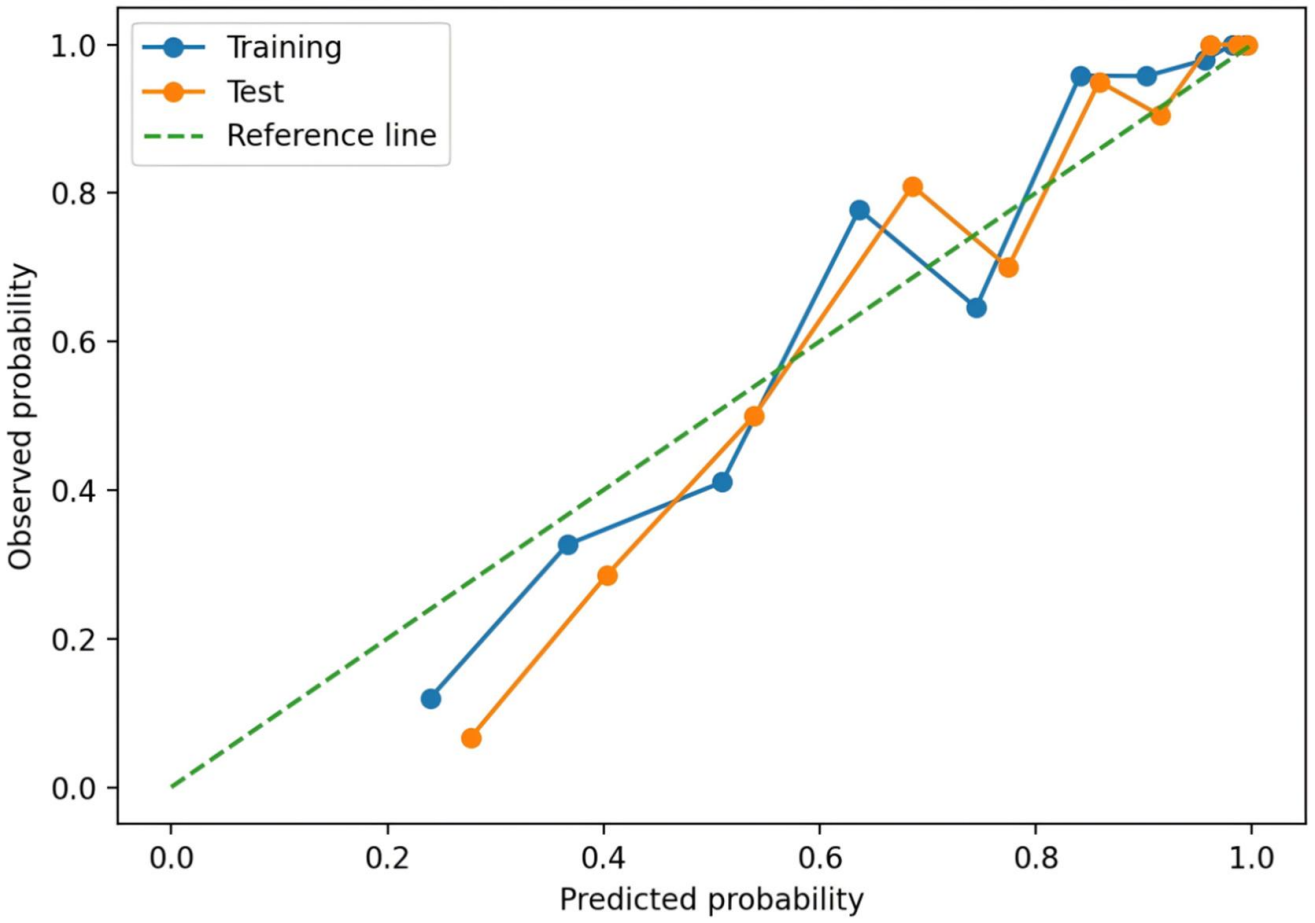
Calibration curves of neonatal septicemia prediction model B in training set and test set. Although model B showed good discrimination, its training-set miscalibration indicates that the sample-based predicted probabilities require recalibration before they can be interpreted as absolute clinical risks in other populations.

Hosmer-Lemeshow test results showed that the *P* values of model A training set and test set were 0.1868 and 0.5416 respectively, and the goodness of fit of the model was better; the *P* values of model B training set and test set were 0.0090 and 0.1414 respectively. Figures 6, 7 were the calibration curves on model A training set and test set, and the calibration curves on model B training set and test set respectively. Among them the horizontal axis was the sample-based probability of predicting neonatal septicemia, and the vertical axis was the observed proportion of neonatal septicemia in the samples. In the figure, the diagonal line was the reference line; curves closer to this line suggested better calibration within the study sample. Model A showed acceptable calibration in both sets, whereas model B showed significant training-set miscalibration (Hosmer-Lemeshow *P* = 0.0090) despite acceptable test-set fit (Figure 8).

## 4 Discussion

This study developed and internally validated two pragmatic diagnostic prediction models for neonatal sepsis, demonstrating that a multivariable approach integrating diverse clinical and maternal factors can achieve useful discriminatory performance. The first model, incorporating readily available maternal history and neonatal clinical examination findings without laboratory tests, showed robust discrimination with an area under the curve exceeding 0.85 in both training and test sets. The second model, which augmented these clinical predictors with two routine laboratory markers, white blood cell count and C-reactive protein, achieved superior diagnostic accuracy, with AUC values above 0.90. The core finding is that while a clinical-only model holds significant utility, particularly in resource-constrained contexts, the incremental value of adding basic inflammatory biomarkers is substantial, moving the model performance into a range often considered excellent for diagnostic tools. This aligns with the persistent clinical challenge wherein neonatal sepsis manifests with nonspecific signs, and no single parameter is sufficiently reliable, necessitating composite assessment strategies (39, 40).

The predictors retained in our final models span several domains consistently implicated in sepsis risk. Maternal factors such as residence area and history of threatened abortion may serve as proxies for access to care, socioeconomic status, or underlying maternal inflammatory states that predispose to adverse neonatal outcomes. For instance, maternal infection and fever are well-established risk factors for early-onset sepsis (41, 42). The inclusion of prenatal antibiotic exposure is particularly noteworthy. While intrapartum antibiotic prophylaxis is standard for preventing Group B Streptococcus disease, broader prenatal antibiotic exposure has been associated with an increased risk of neonatal sepsis, possibly due to microbiome disruption or selection for resistant organisms (43, 44). Our finding aligns with this complex relationship, suggesting that a history of antibiotic use before delivery should be considered in risk stratification, independent of intrapartum prophylaxis. On the neonatal side, clinical signs like temperature instability and depressed neurological status (weak mental response, abnormal primitive reflexes) are classic, albeit nonspecific, indicators of systemic illness and are commonly incorporated into sepsis scores and clinical assessments (28, 45). Feeding status emerged as a strong predictor, corroborating other studies where feeding intolerance or decreased feeding volume were key clinical markers of late-onset sepsis in both term and preterm infants (45, 46).

The weight difference variable bridges nutritional intake, fluid balance, and catabolic state, offering a simple quantitative measure of infant well-being. The administration of antibiotics before the current visit, which could represent treatment for a prior or ongoing infection, logically increases the probability of a sepsis diagnosis, though it may also indicate clinical suspicion that preceded formal evaluation. The most significant performance gain came from incorporating abnormal white blood cell count and elevated CRP. These are among the most widely used laboratory biomarkers in neonatal sepsis diagnostics. C-reactive protein is a classic acute-phase reactant, but its rise can be delayed by 12–24 h after infection onset, and it can be elevated in non-infectious inflammatory conditions (19, 47). White blood cell count and its derivatives, such as the immature-to-total neutrophil ratio, are routine hematological parameters with recognized but imperfect diagnostic characteristics (48). Their individual limitations in sensitivity and specificity are well-documented (26, 40). Our analysis demonstrates that their predictive power is significantly enhanced when contextualized within a broader clinical model. Rather than relying on a single cutoff, the nomogram weighs these values alongside maternal history and clinical exam, potentially mitigating false positives from non-infectious inflammation and false negatives from early sampling.

Our work adds to a growing body of literature seeking to improve neonatal sepsis diagnosis through prediction modeling (49, 50). Numerous scores and calculators exist, with varying predictor sets and intended uses. The widely studied Kaiser Permanente early-onset sepsis calculator, for example, focuses on a specific set of intrapartum maternal risk factors and infant clinical appearance to estimate sepsis risk in neonates ≥34 weeks, primarily aiming to reduce unnecessary antibiotic exposure (10, 51–53). Its success in achieving that goal highlights the value of risk stratification tools (54, 55). In contrast, our models were developed from a hospitalized population spanning both early and late-onset sepsis and incorporated a wider array of variables, including postnatal feeding, pre-admission antibiotics, and detailed neurological signs. This makes them more applicable to the broader diagnostic question faced in a neonatal unit rather than the specific scenario of immediate postnatal management. Other published nomograms share similarities in approach. For instance, a model by Sokou et al. combined clinical signs, laboratory results, and thromboelastometry parameters, achieving excellent discrimination (29). Another nomogram focused on predicting sepsis complicated by purulent meningitis, identifying factors like seizures and abnormal laboratory values (30). These studies, like ours, underscore the trend toward multivariable, context-specific tools rather than universal single biomarkers.

The performance characteristics of our two models suggest a potential future application: a two-tiered diagnostic strategy after external validation and recalibration. Model A, relying solely on historical and clinical examination data, requires no laboratory infrastructure. It demonstrated a specificity above 0.90 in the training set, indicating a strong ability to rule in sepsis when clinical suspicion is high based on these features alone. After external validation, this model could be useful in low-resource settings where laboratory testing is unavailable or delayed, or for initial triage in any setting to identify infants warranting closer assessment while laboratory work is pending (36, 56, 57). Its sensitivity, while moderate, may be sufficient for this first-line screening role when combined with clinical vigilance. Model B, with its superior sensitivity and overall accuracy in this sample, may be more relevant for hospital settings where complete blood count and CRP are routinely available. The significant improvement in AUC from Model A to Model B quantifies the concrete diagnostic benefit of these simple blood tests when interpreted algorithmically. This stratified approach aligns with calls for tools that may support future antibiotic-stewardship workflows after prospective impact evaluation (44, 58, 59).

It is important to acknowledge that several of the strongest individual predictors in both models—including abnormal temperature, weak mental response, primitive reflex abnormalities, abnormal white blood cell count, and elevated C-reactive protein—are already integral components of routine neonatal sepsis evaluation. The incremental value of the nomograms lies not in the novelty of the individual variables but in their systematic, probabilistic integration into a single quantitative risk estimate. In routine clinical practice, the interpretation of these findings is typically subjective and may vary considerably across providers, particularly among less-experienced clinicians. A structured nomogram may improve diagnostic consistency by standardizing how multiple clinical and laboratory signals are weighed against one another. Potential clinical applications include reducing unnecessary blood cultures and empirical antibiotic courses in infants classified as low-risk, facilitating earlier recognition of sepsis in infants whose individual findings are each borderline but collectively reach a high predicted probability, and providing a reproducible decision framework for antibiotic stewardship programs. However, these potential benefits remain hypothetical until evaluated in prospective implementation studies.

A balanced interpretation of our models’ performance is essential. The high AUC values, particularly for Model B, indicate excellent discrimination between sepsis and non-sepsis cases in our cohort. The sensitivity and specificity figures represent a point on the receiver operating characteristic curve chosen by the modeling process; in practice, the cutoff could be adjusted to favor either sensitivity (to avoid missing cases) or specificity (to reduce overtreatment) based on clinical context and risk tolerance. The trade-off is evident: Model B's higher sensitivity (0.864–0.910) comes with a lower specificity (0.750–0.800) compared to Model A's profile. Calibration, which assesses how closely predicted probabilities match observed outcomes, was acceptable for Model A but showed significant weakness for Model B in the training set. This suggests that while Model B ranks patients’ risk accurately, its absolute probability estimates require recalibration before clinical use, particularly because the case-control sampling fraction does not represent the target population prevalence (36). This does not undermine the model's discriminatory utility but highlights a need for potential recalibration before deployment in new populations.

### 4.1 Strengths, limitations, and future directions

Key strengths of this study include the relatively large sample size of confirmed sepsis cases and controls, which provides stability to the estimated coefficients. The predictor selection process, combining univariable screening with LASSO regularization, is a robust method to identify the most informative variables from a broader set while mitigating overfitting. The variables chosen are largely pragmatic, routinely documented in clinical records, enhancing the potential for real-world implementation. The internal validation via a hold-out test set provides a reasonable, though preliminary, estimate of model performance on unseen data from the same institution.

Several important limitations must be acknowledged. The single-center, retrospective design is a primary constraint. Data were extracted from medical records, which may be incomplete or inconsistently recorded, introducing potential information bias. The control group, comprising neonates with non-septic infections or no infection, represents a pragmatic clinical comparison but introduces heterogeneity; the model performance might differ if tested against strictly healthy newborns. Selection bias is possible, as the inclusion relied on clinical suspicion leading to a sepsis workup. In addition, several Model A predictors, including abnormal temperature, weak mental response, and abnormal primitive reflexes, overlap with clinical manifestations used in case ascertainment; this may introduce diagnostic-review bias, although the overlap is less direct than the incorporation of WBC and CRP in Model B. The case-control design also means that nomogram probabilities, calibration curves, and Brier-type calibration summaries reflect the sampled case fraction and should not be interpreted as population absolute risks without recalibration. The lack of external validation is a critical gap. Models often perform less well in different populations with varying patient demographics, clinical practices, and pathogen prevalence (18, 60). The generalizability of our nomograms, particularly the variable coefficients and optimal cutoffs, to other hospitals, regions, or healthcare systems is unknown. Furthermore, the models do not distinguish between early- and late-onset sepsis, which have different etiologies, risk factors, and common pathogens (61, 62); a unified model may not be optimal for both scenarios. Additionally, the composition of the control group warrants consideration: because the control group included both non-septic infection and non-infected neonates, the overall model discrimination may overestimate performance relative to the harder clinical task of distinguishing sepsis from other non-septic infections. As shown in Supplementary Table S2, when controls were restricted to non-septic infectious cases only, AUC values decreased (model A: 0.816; model B: 0.889), a finding that reflects the greater diagnostic challenge in this subgroup. Furthermore, the proportion of clinically diagnosed sepsis cases (61.3%) introduces the possibility of outcome misclassification, because clinically diagnosed sepsis has lower diagnostic specificity than culture-confirmed sepsis. The postnatal age distribution in this cohort (median 5 days, IQR 2–12 days) should also be considered when interpreting laboratory marker performance, as several neonatal biomarkers exhibit significant age-dependent physiological variation. With respect to temperature assessment, axillary measurement rather than rectal measurement was used, which may have introduced some misclassification of febrile status given that rectal temperature remains the reference standard in neonates. The reliance on a single 7:3 train-test split for internal validation, rather than more robust methods such as bootstrapping or repeated cross-validation, may have produced partition-dependent performance estimates.

Future research must prioritize external validation in independent, preferably multicenter, cohorts (36, 63). This step is crucial to assess transportability and identify any need for model updating or recalibration. Prospective implementation studies are the logical next step to evaluate the real-world clinical impact of using these nomograms. Such studies should measure outcomes like antibiotic initiation rates, duration of therapy, time to appropriate treatment, and clinical outcomes, comparing guided management to usual care (59, 64). Investigating the integration of novel biomarkers with higher specificity, such as presepsin or interleukin-6, could be a fruitful avenue to further enhance performance beyond CRP and WBC, though cost and availability remain barriers (65–67). Finally, after external validation, translating the nomogram into an electronic health record-embedded clinical decision support tool or a simple mobile application could facilitate prospective evaluation. In conclusion, this study provides two internally validated diagnostic models that synthesize multifaceted clinical information into structured, sample-based risk estimates for neonatal sepsis. The findings reinforce the superiority of integrated models over isolated parameters and propose a pragmatic framework for stratified diagnosis adaptable to different healthcare resources.

## 5 Conclusions

This study develops and internally validates two practical nomogram-based diagnostic models for neonatal sepsis using routinely available maternal, clinical, and laboratory data from a single-center retrospective cohort. Both models demonstrate useful discriminatory ability, while the model incorporating white blood cell count and C-reactive protein shows stronger overall diagnostic performance than the clinical model alone. The findings support the feasibility of structured multivariable risk estimation as an adjunct to early neonatal sepsis recognition, particularly when definitive microbiological confirmation is delayed or limited in sensitivity. At the same time, the results indicate that model performance should be interpreted cautiously because calibration is not uniformly optimal and validation remains internal only. Overall, we provide a pragmatic framework for bedside risk-estimation research in neonatal sepsis and offer a basis for further refinement. External validation, recalibration in independent populations, and prospective evaluation in diverse neonatal care settings are needed before routine clinical implementation can be recommended.

## Data Availability

The raw data supporting the conclusions of this article will be made available by the authors, without undue reservation.
