# Supplementary material for: Construction and validation of neonatal sepsis diagnostic nomograms: a single-site retrospective study
Source: Front Pediatr. 2026 Jul 28;14:1841983. doi: 10.3389/fped.2026.1841983 (PMC13457390; doi:10.3389/fped.2026.1841983)
Supplement: Supplementary file 1 [file Datasheet1.pdf]

Supplementary Table S1. Full logistic regression model specifications

Predictor coding: maternal residence (1=urban, 2=rural); threatened abortion, prenatal antibiotics, abnormal temperature, weak mental response, abnormal primitive reflex, pre-visit antibiotics, abnormal WBC, elevated CRP (0=no, 1=yes); breastfeeding (0=any breast milk, 1=no breast milk); visit-vs-birth weight difference (1=zero/positive, 2=negative).

**Predictor**	**Model A coefficient (SE)**	**Model A OR (95% CI)**	**Model B coefficient (SE)**	**Model B OR (95% CI)**
Intercept	-2.184 (0.412)	—	-3.026 (0.468)	—
Maternal residence (rural vs urban)	0.742 (0.171)	2.10 (1.50-2.93)	0.718 (0.169)	2.05 (1.47-2.86)
Threatened abortion	2.498 (0.982)	12.18 (1.78-83.4)	2.381 (0.971)	10.81 (1.61-72.6)
Breastfeeding (no vs any breast milk)	0.649 (0.181)	1.91 (1.34-2.73)	0.612 (0.178)	1.84 (1.30-2.61)
Prenatal antibiotics	1.258 (0.481)	3.52 (1.37-9.03)	1.201 (0.476)	3.32 (1.31-8.42)
Infant temperature abnormal	1.160 (0.221)	3.19 (2.07-4.91)	1.118 (0.218)	3.06 (2.00-4.68)
Weak mental response	1.813 (0.325)	6.15 (3.25-11.6)	1.742 (0.319)	5.71 (3.06-10.7)
Primitive reflex abnormal	2.612 (0.583)	13.6 (4.35-42.7)	2.498 (0.571)	12.2 (3.98-37.4)
Pre-visit antibiotics	1.870 (0.345)	6.49 (3.30-12.8)	1.804 (0.338)	6.07 (3.14-11.7)
Visit-vs-birth weight	0.412 (0.158)	1.51	0.398 (0.156)	1.49

difference		(1.11-2.06)		(1.10-2.02)
Abnormal WBC	—	—	3.045 (0.512)	21.0 (7.69-57.4)
Elevated CRP	—	—	1.908 (0.233)	6.75 (4.27-10.7)

Supplementary Table S2. Sensitivity and stratified discrimination analyses

**Analysis**	**Model A test-set AUC (95% CI)**	**Model B test-set AUC (95% CI)**
Full cohort	0.852 (0.802-0.902)	0.923 (0.891-0.955)
Culture-confirmed cases only	0.831 (0.761-0.901)	0.876 (0.812-0.940)
Clinically diagnosed cases only	0.864 (0.801-0.927)	0.941 (0.901-0.981)
EOS subgroup	0.861 (0.791-0.931)	0.918 (0.862-0.974)
LOS subgroup	0.841 (0.761-0.921)	0.901 (0.841-0.961)
Model B rebuilt without WBC/CRP	—	0.867 (0.807-0.927)
Controls restricted to non-septic infection	0.816 (0.736-0.896)	0.889 (0.829-0.949)
Controls restricted to no infection	0.871 (0.801-0.941)	0.948 (0.908-0.988)
